# Group B Streptococcal Infection of the Choriodecidua Induces Dysfunction of the Cytokeratin Network in Amniotic Epithelium: A Pathway to Membrane Weakening

**DOI:** 10.1371/journal.ppat.1003920

**Published:** 2014-03-06

**Authors:** Jeroen P. Vanderhoeven, Craig J. Bierle, Raj P. Kapur, Ryan M. McAdams, Richard P. Beyer, Theo K. Bammler, Federico M. Farin, Aasthaa Bansal, Min Spencer, Mei Deng, Michael G. Gravett, Craig E. Rubens, Lakshmi Rajagopal, Kristina M. Adams Waldorf

**Affiliations:** 1 Department of Obstetrics & Gynecology, University of Washington, Seattle, Washington, United States of America; 2 Center for Childhood Infections and Prematurity Research, Seattle Children's Research Institute, Seattle, Washington, United States of America; 3 Departments of Pathology, Seattle Children's and University of Washington, Seattle, Washington, United States of America; 4 Department of Pediatrics, University of Washington, Seattle, Washington, United States of America; 5 Department of Environmental and Occupational Health Sciences, University of Washington, Seattle, Washington, United States of America; 6 Department of Biostatistics, University of Washington, Seattle, Washington, United States of America; 7 Center on Human Development and Disability, University of Washington, Seattle, Washington, United States of America; 8 Global Alliance to Prevent Prematurity & Stillbirth, Seattle, Washington, United States of America; University of California, San Francisco, United States of America

## Abstract

Early events leading to intrauterine infection remain poorly defined, but may hold the key to preventing preterm delivery. To determine molecular pathways within fetal membranes (chorioamnion) associated with early choriodecidual infection that may progress to preterm premature rupture of membranes (PPROM), we examined the effects of a Group B Streptococcus (GBS) choriodecidual infection on chorioamnion in a nonhuman primate model. Ten chronically catheterized pregnant monkeys (*Macaca nemestrina*) at 118–125 days gestation (term = 172 days) received choriodecidual inoculation of either GBS (n = 5) or saline (n = 5). Cesarean section was performed in the first week after GBS or saline inoculation. RNA extracted from chorioamnion (inoculation site) was profiled by microarray. Single gene, Gene Set, and Ingenuity Pathway Analysis results were validated using qRT-PCR (chorioamnion), Luminex (amniotic fluid, AF), immunohistochemistry, and transmission electron microscopy (TEM). Despite uterine quiescence in most cases, significant elevations of AF cytokines (TNF-α, IL-8, IL-1β, IL-6) were detected in GBS versus controls (p<0.05). Choriodecidual infection resolved by the time of cesarean section in 3 of 5 cases and GBS was undetectable by culture and PCR in the AF. A total of 331 genes were differentially expressed (>2-fold change, p<0.05). Remarkably, GBS exposure was associated with significantly downregulated expression of multiple cytokeratin (CK) and other cytoskeletal genes critical for maintenance of tissue tensile strength. Immunofluorescence revealed highly significant changes in the CK network within amniocytes with dense CK aggregates and retraction from the cell periphery (all p = 0.006). In human pregnancies affected by PPROM, there was further evidence of CK network retraction with significantly shorter amniocyte foot processes (p = 0.002). These results suggest early choriodecidual infection results in decreased cellular membrane integrity and tensile strength via dysfunction of CK networks. Downregulation of CK expression and perturbations in the amniotic epithelial cell intermediate filament network occur after GBS choriodecidual infection, which may contribute to PPROM.

## Author Summary

Group B Streptococcus (GBS) is one cause of preterm birth, stillbirth, and fetal brain injury. GBS is present in the vagina and is thought to ascend into the uterus of some women where it can cause placental inflammation and preterm birth. Understanding the earliest events in the placenta that lead to preterm birth is elusive in humans, because the placenta cannot be studied until after birth. Here, we use a nonhuman primate model to show that an early GBS infection can damage the structural support of the fetal membranes, specifically the cytokeratin network in the epithelium of the amnion (one part of the membranes). Next, we obtained human placentas to show that this cytokeratin network was also damaged in human patients that had preterm premature rupture of the membranes, a major cause of preterm birth. Our work is important in understanding why fetal membranes may rupture prematurely, which may lead to early interventions to prevent membrane damage after placental infection and preterm birth.

## Introduction

Preterm premature rupture of membranes (PPROM) occurs in 1–2% of all pregnancies, but complicates 30% of all preterm deliveries [1]. The majority of women (70%) with PPROM deliver within 24 hours after membrane rupture. The fetal membranes (chorioamnion) are composed of two membranes called the amnion and chorion that enclose the amniotic cavity. Inflammation of the chorioamnion and within the amniotic fluid is thought to play a major role in the pathogenesis of premature rupture resulting in preterm delivery. Infection-associated preterm labor is commonly characterized by elevated amniotic fluid cytokine levels in women and animal models of preterm birth [2], [3], [4], [5], [6], In addition to mediating inflammation, cytokines have also been associated with increased collagen remodeling and, ultimately, biophysical weakening of the fetal membranes or chorioamnion *in vitro* [7]. Unfortunately, current therapies to prevent preterm delivery following membrane rupture are of limited efficacy. In the setting of PPROM attributed to infection, there are currently no therapeutic alternatives to immediate delivery [8]. Beyond the prevention of urogenital infection, clinically useful biomarkers for preterm membrane rupture have not yet been identified to aid in meaningful prevention efforts [9].

The underlying pathogenesis of PPROM is thought to be due to a combination of physical stresses and biochemical weakening of the chorioamnion. In association with infection, investigation of PPROM has mainly focused on the catabolic degradation of collagen mediated by matrix metalloproteinases (MMP) with some studies of other pathways including apoptosis and oxidative stress [10], [11], [12], [13], [14]. In normal pregnancy, biochemical weakening of the membranes occurs in the last few weeks prior to the onset of labor at term (between 37–41 weeks). However, under pathologic conditions of infection and inflammation, early MMP activation may lead to chorion thinning, premature membrane rupture and preterm birth [12], [15], [16].

We hypothesized that in addition to MMP degradation of extracellular collagen (mainly in the chorion), pathways targeting the intracellular cytoskeleton of the amniotic epithelium would be important in creating vulnerability for PPROM. This hypothesis was driven by the observation that the chorion, while thicker, is weaker than the amnion suggesting that biomechanical failure of structural components unique to amnion may be critical for membrane rupture [17]. To investigate early factors involved in fetal membrane injury, we performed a secondary analysis of data obtained from a previously described chronically catheterized pregnant nonhuman primate model (pigtail macaque; *Macaca nemestrina*), which shares many important features with human pregnancy [18]. We infused Group B *Streptococcus*, an organism associated with preterm birth [19], into the choriodecidual space via a catheter placed between the uterine muscle and membranes (external to the amniotic fluid) overlying the lower uterine segment.

Our nonhuman primate model had previously demonstrated that a transient GBS infection located within the choriodecidua can induce a cytokine response within the chorioamnion and amniotic fluid (Table S1). This response was associated with labor in two of the GBS animals. Evidence of chorioamnionitis was noted on placental histology of only two of the five GBS animals whereas the remaining animals had normal histology (Figure S1, representative images). Amniotic fluid remained culture negative in all animals. Using samples obtained from this model, we describe, for the first time, molecular pathways that are activated and disrupted in the chorioamnion associated with cytokine elevations induced by a GBS choriodecidual infection.

## Results

We previously reported uterine contraction, microbial culture, and cytokine (amniotic fluid and fetal plasma) results in our model of early choriodecidual infection in association with *in utero* fetal lung injury (Table S1, Fig. S1) [20].

### Single Gene Analysis

To identify early genomic events following a GBS choriodecidual infection, we performed microarray analysis of chorioamnion from the site of inoculation. Of the differentially expressed genes, a subset is shown in Table 1 and the entire set meeting at least 2.0 fold criteria (331 genes) and a heatmap (603 probesets, 331 genes) is available in supplementary material (Table S2, Fig. S2).

**Table 1:** Selected examples of most differentially regulated single genes.

Description	Symbol	log2 fold change
Similar to CK 24	LOC701667	−5.90
CK 3	KRT3	−5.31
Similar to CK 6A	LOC718942	−5.27
Similar to CK 6A	LOC718942	−5.25
Zinc finger, BED-type containing 2	ZBED2	−4.48
Solute carrier family 27, member 6	SLC27A6	−4.24
Similar to Laminin 5 beta 3	LOC717602	−4.10
Laminin, gamma 2	LAMC2	−3.95
Similar to CK 16	LOC707236	−3.85
Similar to CK 6A	LOC718942	−3.82
Similar to breast cancer membrane protein 11	LOC714517	−3.80
Similar to Desmocollin 2 isoform A (DSC2A)	LOC712942	−3.79
Similar to CK 14	LOC703932	−3.75
Platelet/endothelial cell adhesion molecule	PECAM1	1.85
Colony stimulating factor 3 receptor	CSF3R	1.87
Chemokine ligand 12	CXCL12	1.88
Myosin, heavy chain 11, smooth muscle	MYH11	1.91
Colony stimulating factor 2 receptor, beta	CSF2RB	1.94
Similar to microfibrillar-associated protein 4	LOC709521	1.95
Similar to DCP2 decapping enzyme	LOC694225	1.99
Similar to DCP2 decapping enzyme	LOC694225	2.04
Fatty acid binding protein 4, adipocyte	FABP4	2.15
Similar to DCP2 decapping enzyme	LOC694225	2.18
Matrix metallopeptidase 1	MMP1	2.28
Myosin, heavy chain 11, smooth muscle	MYH11	2.32
Similar to HspB1	HSPB1	2.42
Placenta-specific 8	PLAC8	2.57

Genes significantly downregulated by choriodecidual GBS exposure included multiple cytokeratins (CK) such as *CK3, CK 6A, CK7, CK8, CK 14, CK15, CK 16*, *CK 19*, and *CK 24* (range of log_2_ fold change: −1.60 to −5.90; all p<0.05). Many collagens, collagen precursors and collagen binding proteins were also downregulated including *COL1A2, COL7A1, COL5A1*, and *LUM* (lumican; range of log_2_ fold change: −1.73 to −3.52; all p<0.05). Many genes expressing components of the intracellular matrix were significantly downregulated including laminins (beta-3 chain precursor and gamma-2), desmocollin 2 (*DSC 2*), and desmoplakin (*DSP*). Examples of genes significantly upregulated include colony stimulating factor 3 receptor (*CSF3R*) and chemokine (C-X-C motif) ligand 12 (*CXCL12*) involved in inflammation, matrix metalloproteinase 1 (*MMP1*), and a gene similar to DCP2 decapping enzyme, which increases mRNA degradation and negatively controls the innate immune response. These data indicate significant downregulation of cytostructural support elements in the setting of increased inflammation and MMP gene expression.

Both a three-factor analysis *(factor 1: GBS with chorioamnionitis, factor 2: GBS no chorioamnionitis, factor 3: saline)* and principal component analysis failed to identify differences between the two samples in the GBS-exposed group with histologic evidence of chorioamnionitis and the remaining GBS-exposed animals without chorioamnionitis with the exception of one gene (Fig. S3). In the three-factor analysis, the gene expression of key genes or proteins discussed in our manuscript (i.e. TNF-α, IL-1β, IL-6, cytokeratins, *PLAC8*, *CXCL12*, *PECAM1*, *LAMB3*, *SCEL*, *DSG2*) had the same up- or downregulation regardless of chorioamnionitis with the exception of *MMP1*. *MMP1* gene expression was upregulated in GBS animals without chorioamnionitis, but similar to controls in the two animals with chorioamnionitis.

### Gene Set Analysis (GSA)

**Figure 1:**
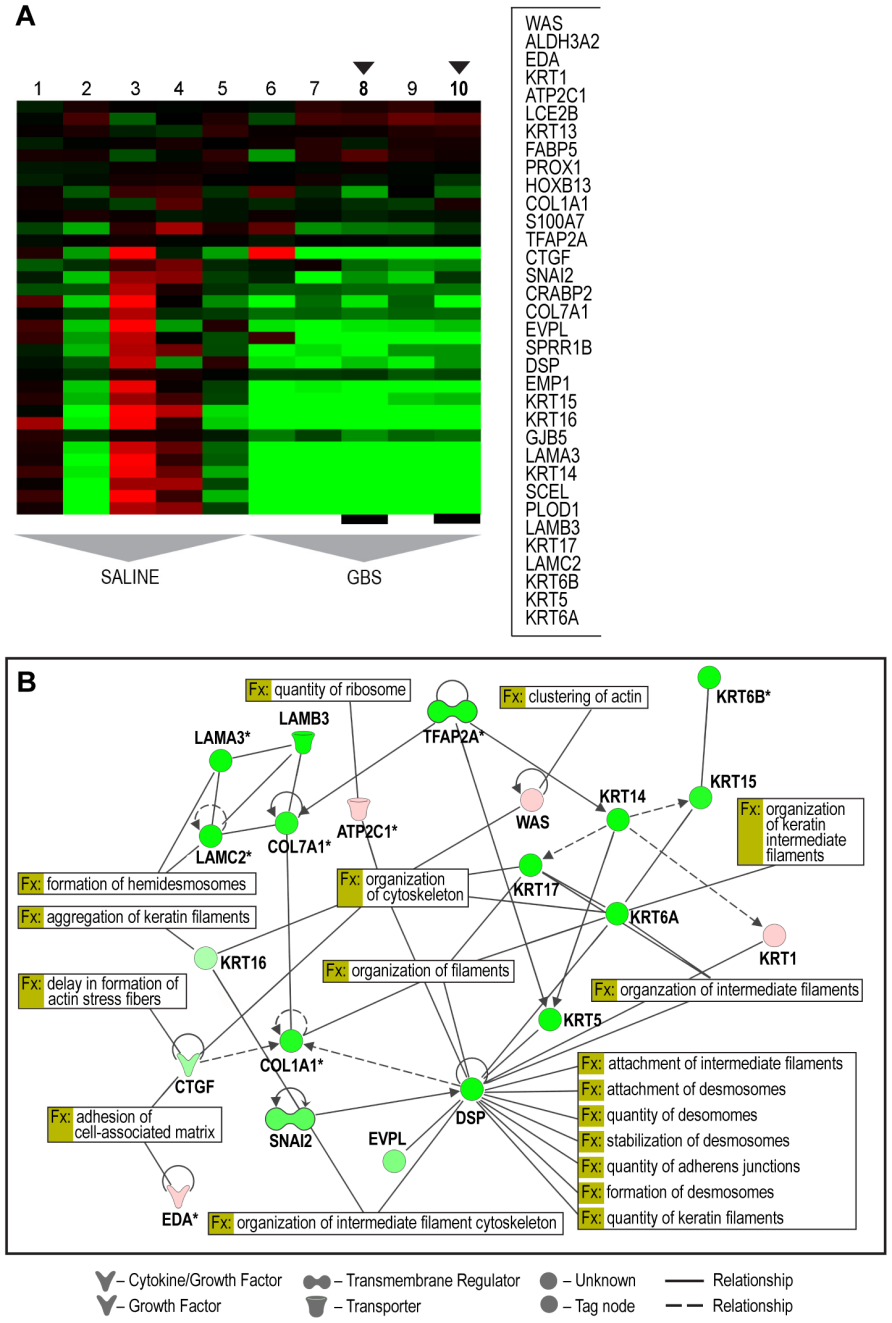
Selected heatmap and paired Ingenuity Pathway Analysis diagrams demonstrating downregulation across cytostructural components including multiple cytokeratins, laminins, and collagens (panel A: C4 Module 153; panel B: IPA diagram). Genes shown were filtered for interactions reported in humans only. Direct and indirect interactions are shown by solid lines and dashed lines respectively. Green indicates gene downregulation and red depicts upregulation. Color intensity represents the average of log2 fold change with brighter colors representing a more significant difference between GBS and controls. Symbols for each molecule are presented according to molecular functions and type of interactions. Functional assignations attributed by IPA software. An asterisk indicates that there were multiple probesets for the same gene on the Affymetrix array. The black arrows indicate the GBS cases with chorioamnionitis.

To further explore the relationships between differentially expressed single genes, we identified gene sets and pathways with concordant changes in expression using GSA [21]. Gene sets downregulated following GBS exposure were frequently related to epidermis development, keratinization, negative regulation of actin filament polymerization, hemidesmosome assembly, intermediate filament-based processes, intermediate filament organization, and the intermediate filament cytoskeleton (all p = 0.006, Table 2, Table S3 and Fig. 1). Gene sets enriched after GBS exposure were frequently related to immune activation and programmed cell death including myeloid leukocyte differentiation, apoptotic DNA fragmentation, protein monoubiquitination, T cell homeostasis, and positive regulation of macrophage differentiation (all p<0.002, Tables 3 and S4). GSA indicated that the large-scale downregulation of cytokeratins evident in the single gene analysis was related to the negative regulation of many other genes necessary for intermediate filament function. Upregulation of inflammatory and apoptotic pathways in our data is consistent with histologic studies of the membrane rupture site in women with PPROM [22], [23], [24].

**Table 2:** Selected examples of most downregulated gene sets after GBS exposure relative to the control group.

Gene set description	Gene set length	Gene Ontology ID	p value
**Biological process**			
morphogenesis of an epithelium	23	GO:0002009	0.006
ectoderm development	18	GO:0007398	0.006
epidermis development	89	GO:0008544	0.006
positive regulation of peptidase activity	12	GO:0010952	0.006
peptide cross-linking	22	GO:0018149	0.006
galactose catabolic process	7	GO:0019388	0.006
negative regulation of actin filament polymerization	7	GO:0030837	0.006
negative regulation of epithelial cell differentiation	6	GO:0030857	0.006
keratinization	26	GO:0031424	0.006
hemidesmosome assembly	12	GO:0031581	0.006
cell junction assembly	74	GO:0034329	0.006
intermediate filament-based process	7	GO:0045103	0.006
intermediate filament organization	15	GO:0045109	0.006
**Molecular function**			
SNARE binding	19	GO:0000149	0.006
structural constituent of cytoskeleton	93	GO:0005200	0.006
thrombin receptor activity	5	GO:0015057	0.006
peptidase activator activity	10	GO:0016504	0.006
oxidoreductase activity, acting on single donors with incorporation of molecular oxygen, incorporation of two atoms of oxygen	77	GO:0016702	0.006
oxidoreductase activity, acting on paired donors, with incorporation or reduction of molecular oxygen, 2-oxoglutarate as one donor, and incorporation of one atom each of oxygen into both donors	33	GO:0016706	0.006
L-ascorbic acid binding	21	GO:0031418	0.006
protein kinase A catalytic subunit binding	10	GO:0034236	0.006
heterotrimeric G-protein binding	6	GO:0032795	0.01
chemorepellent activity	5	GO:0045499	0.011
neurotransmitter transporter activity	5	GO:0005326	0.012
MHC class II protein binding	5	GO:0042289	0.012
structural molecule activity	223	GO:0005198	0.013
AMP-activated protein kinase activity	7	GO:0004679	0.015
oxidoreductase activity, acting on paired donors, with incorporation or reduction of molecular oxygen	47	GO:0016705	0.015
**Cellular component**			
sarcoplasm	6	GO:0016528	0.006
rough endoplasmic reticulum membrane	7	GO:0030867	0.006
pseudopodium	14	GO:0031143	0.006
intermediate filament cytoskeleton	45	GO:0045111	0.006
flagellar axoneme	9	GO:0035086	0.007
platelet alpha granule lumen	46	GO:0031093	0.009
cell periphery	28	GO:0071944	0.01
AMP-activated protein kinase complex	7	GO:0031588	0.012
pyruvate dehydrogenase complex	7	GO:0045254	0.014
photoreceptor outer segment	38	GO:0001750	0.016
basement membrane	91	GO:0005604	0.016
microtubule associated complex	34	GO:0005875	0.016
costamere	20	GO:0043034	0.019
intermediate filament	97	GO:0005882	0.02
acrosomal membrane	11	GO:0002080	0.021

**Table 3:** Selected examples of most upregulated gene sets after GBS exposure relative to the control group.

Gene set description	Gene set length	Gene Ontology ID	p value
**Biological process**			
myeloid leukocyte differentiation	5	GO:0002573	<0.002
apoptotic DNA fragmentation	25	GO:0006309	<0.002
protein monoubiquitination	17	GO:0006513	<0.002
pinocytosis	5	GO:0006907	<0.002
regulation of cellular pH	5	GO:0030641	<0.002
T cell receptor V(D)J recombination	5	GO:0033153	<0.002
response to laminar fluid shear stress	10	GO:0034616	<0.002
T cell homeostasis	21	GO:0043029	<0.002
regulation of bone resorption	7	GO:0045124	<0.002
positive regulation of erythrocyte differentiation	16	GO:0045648	<0.002
vagina development	10	GO:0060068	<0.002
neural precursor cell proliferation	6	GO:0061351	<0.002
erythrocyte maturation	11	GO:0043249	0.002
positive regulation of survival gene product expression	12	GO:0045885	0.002
positive regulation of macrophage differentiation	8	GO:0045651	0.004
gland development	5	GO:0048732	0.004
**Molecular function**			
Rho GTPase activator activity	24	GO:0005100	0.006
ARF GTPase activator activity	25	GO:0008060	0.006
cytokine binding	20	GO:0019955	0.006
RS domain binding	7	GO:0050733	0.006
telomeric DNA binding	11	GO:0042162	0.007
Rac guanyl-nucleotide exchange factor activity	8	GO:0030676	0.009
eukaryotic cell surface binding	27	GO:0043499	0.009
SH3/SH2 adaptor activity	51	GO:0005070	0.01
oxidoreductase activity, acting on the CH-CH group of donors, NAD or NADP as acceptor	6	GO:0016628	0.01
GTPase activator activity	183	GO:0005096	0.011
organic anion transmembrane transporter activity	15	GO:0008514	0.011
sodium-independent organic anion transmembrane transporter activity	5	GO:0015347	0.011
L-amino acid transmembrane transporter activity	7	GO:0015179	0.013
chromo shadow domain binding	6	GO:0070087	0.013
scavenger receptor activity	45	GO:0005044	0.014
**Cellular component**			
nuclear chromosome	29	GO:0000228	0.006
integral to nuclear inner membrane	5	GO:0005639	0.006
nuclear lamina	9	GO:0005652	0.006
clathrin coated vesicle membrane	13	GO:0030665	0.006
clathrin coat	9	GO:0030118	0.011
T cell receptor complex	12	GO:0042101	0.014
intercellular bridge	6	GO:0045171	0.015
phagocytic vesicle	14	GO:0045335	0.015
extracellular vesicular exosome	7	GO:0070062	0.019
heterogeneous nuclear ribonucleoprotein complex	20	GO:0030530	0.022
IkappaB kinase complex	10	GO:0008385	0.023
spindle midzone	12	GO:0051233	0.024
phosphatidylinositol 3-kinase complex	10	GO:0005942	0.027
chromocenter	8	GO:0010369	0.028
actin filament	44	GO:0005884	0.03

### Ingenuity Pathway Analysis (IPA)

To place the genomics findings of cytostructural downregulation, apoptosis, and inflammation within a visual construct, IPA's Core Analysis feature was used to map functional networks of relevant genes. The top five molecular and cellular function pathways identified by IPA were cell death and survival, cellular movement, cellular growth and proliferation, cellular development, and cell morphology (Table 4). IPA analysis also has the capability to predict activation states of transcriptional regulators based on the activation or suppression of downstream genes. The top five transcription factors predicted to be associated with changes in genes expression were *TGFB1* (transforming growth factor, beta 1), *TNF* (tumor necrosis factor), lipopolysaccharide, *TP53* (tumor protein 53; p53), and *HIF1A* (Hypoxia-inducible factor 1-alpha). P53 regulates expression of genes involved in both growth and apoptosis through activation of gene transcription and has a known role in activation of some CK (e.g. *CK8*) and collagen genes [25], [26].

**Table 4:** Ingenuity Pathway Analysis summary.

*Functional Analysis of a Network* *		
*Diseases and Disorders*	Number of Molecules	p value
Cancer	326	4.45×10^−18^–8.90×10^−4^
Endocrine System Disorders	84	1.57×10^−11^–3.45×10^−4^
Reproductive System Disease	146	1.57×10^−11^–6.19×10^−4^
Gastrointestinal Disease	135	6.35×10^−9^–5.07×10^−4^
Dermatological Diseases and Conditions	126	6.96×10^−8^–8.90×10^−4^
***Molecular and Cellular Functions***		
Cell Death and Survival	271	6.57×10^−16^–8.80×10^−4^
Cellular Movement	196	4.41×10^−15^–8.05×10^−4^
Cellular Growth and Proliferation	271	8.84×10^−12^–9.30×10^−4^
Cellular Development	265	1.25×10^−11^–9.30×10^−4^
Cell Morphology	152	2.37×10^−8^–6.19×10^−4^
***Physiological Systems***		
Cardiovascular System Development and Function	144	1.06×10^−11^–7.32×10^−4^
Hematologic System Development and Function	188	1.25×10^−11^–9.30×10^−4^
Lymphoid Tissue Structure and Development	98	1.22×10^−9^–7.32×10^−4^
Tumor Morphology	84	1.29×10^−9^–7.47×10^−4^
Organismal Development	166	1.48×10^−9^–8.65×10^−4^

*Canonical Pathway Analysis* **		
Top Canonical Pathways	Ratio	p value
Glioblastoma Multiforme Signaling	24/164	1.33×10^−7^
Role of Macrophages, Fibroblasts, and Endothelial Cells in Rheumatoid Arthritis	34/332	9.04×10^−7^
Macropinocytosis Signaling	13/76	2.67×10^−5^
Colorectal Cancer Metastasis Signaling	27/258	2.70×10^−5^
Sphingosine-1-phosphate Signaling	15/120	1.64×10^−4^

*Transcription Factor Analysis* ***		
Top Transcription Factors	Predicted Activation State	p-value of overlap
TGFB1	Not specified	3.34×10^−15^
TNF	Not specified	3.34×10^−15^
Lipopolysaccharide	Not specified	3.34×10^−15^
TP53	Not specified	3.34×10^−14^
HIF1A	Inhibited	3.34×10^−13^

*The Functional Analysis of a Network identified biological functions and/or diseases that were most significant to the molecules in the network using a right-tailed Fisher's exact test.

**Canonical Pathway Analysis identified pathways from the IPA library that were most significant to the data set.

Significance of the association was measured in two ways: (1) as the ratio of the number of molecules from the focus gene set that map to the pathway to the total number of molecules that map to the canonical pathway and (2) using Fisher's exact test.

***Transcription factor analysis is based on prior knowledge of expected effects between transcription factors and their target genes stored in the IPA library.

The overlap p-value measures whether there is a statistically significant overlap between the dataset genes and the genes regulated by a transcription factor using Fisher's Exact Test.

### Validation of cDNA Microarray by Quantitative RT-PCR

**Figure 2:**
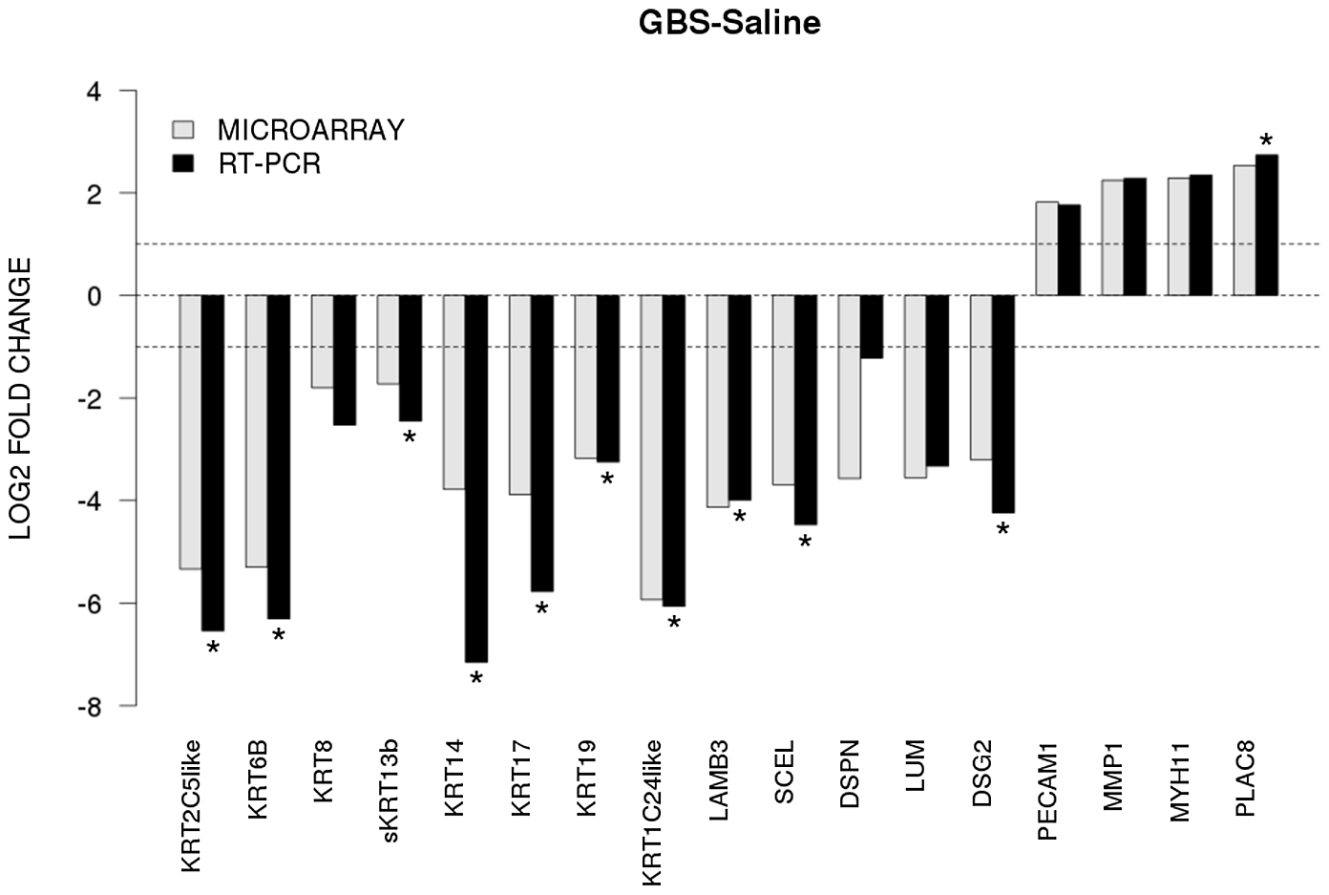
The x-axis represents individual genes and the y-axis fold-change in expression by microarray (gray bars) or qRT-PCR (black bars). Genes that were significantly up- or downregulated by qRT-PCR between GBS and controls are indicated by an asterisk (two-sided t-test, p<0.05). All genes shown were also significant in the unadjusted microarray analysis.

To validate our microarray data, we performed quantitative RT-PCR on 17 genes of interest. We directly compared levels of gene expression obtained with amplified RNA samples using GAPDH expression as a control for input cDNA. Concordance between mRNA generated microarray data and the quantitative RT-PCR data was noted in all samples (Fig. 2). There was a significant difference between GBS and controls for CK 5, 6A, 14, 15, 17, 19, and 24, laminin beta 3 (*LAMB3*), sciellin (*SCEL*), and desmoglein 2 (*DSG2*) (all p<0.05).

### Immunofluorescence and Confocal Microscopy

**Figure 3:**
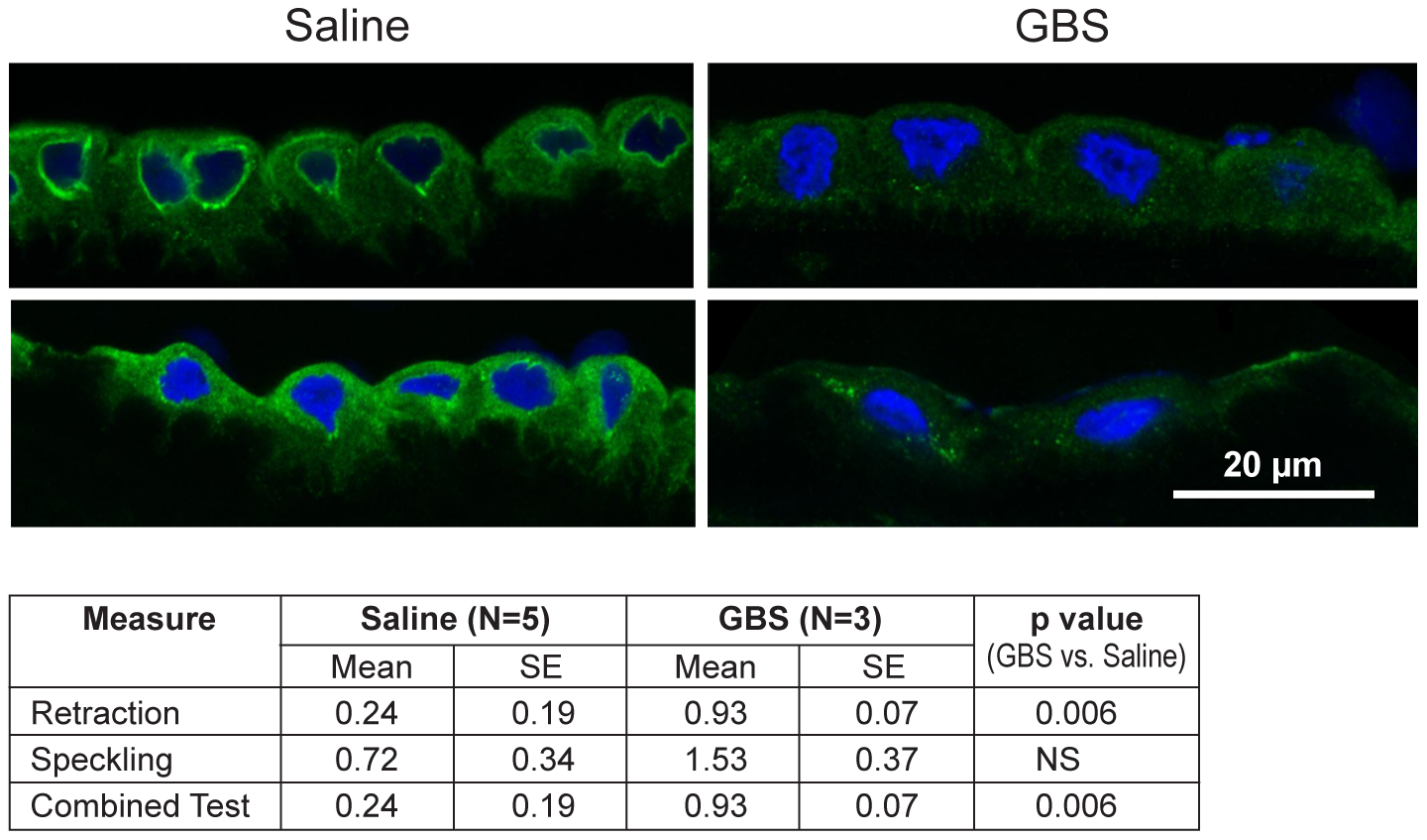
CK-6 immunostained samples of chorioamnion were imaged by confocal microscopy with representative images of saline controls and GBS cases. CK retraction from the cellular periphery was significantly associated with GBS exposure (p = 0.006) as was the combined test of both retraction and speckling (p = 0.006).

Next, we performed immunofluorescence and confocal microscopy to localize CK distribution within the chorioamnion and identify whether morphologic changes typical of CK network dysfunction and depletion were present within the amniotic epithelium. We chose to investigate CK6, because it was significantly downregulated within the microarray data and is expressed in amniotic epithelium. Similar to prior studies, the distribution of CK6 appeared limited to amniotic epithelium, myofibroblasts and macrophages within the extracellular matrix of the chorioamnion as well as the chorion [27], [28]. When examining the amniotic epithelial cells, no difference in overall CK6 content was observed between the GBS-treated group and controls, which was not unexpected as CK intracellular pools remain after *de novo* production has stopped (data not shown). Samples were then scored for speckling or retraction of CK6 from the cellular periphery; speckling is indicative of aggregated partially degraded CK proteins (aggresomes), whereas retraction reflects a lack of CK synthesis in the periphery [29], [30]. The distribution of CK6 immunofluorescence across the amniotic epithelial cell layer was more centralized in the GBS cases than controls; controls had more diffuse staining than GBS cases (Fig. 3). CK retraction from the cellular periphery was significantly associated with GBS exposure (p = 0.006) as was the combined test of both retraction and speckling (p = 0.006) by logistic regression.

### Transmission Electron Microscopy

**Figure 4:**
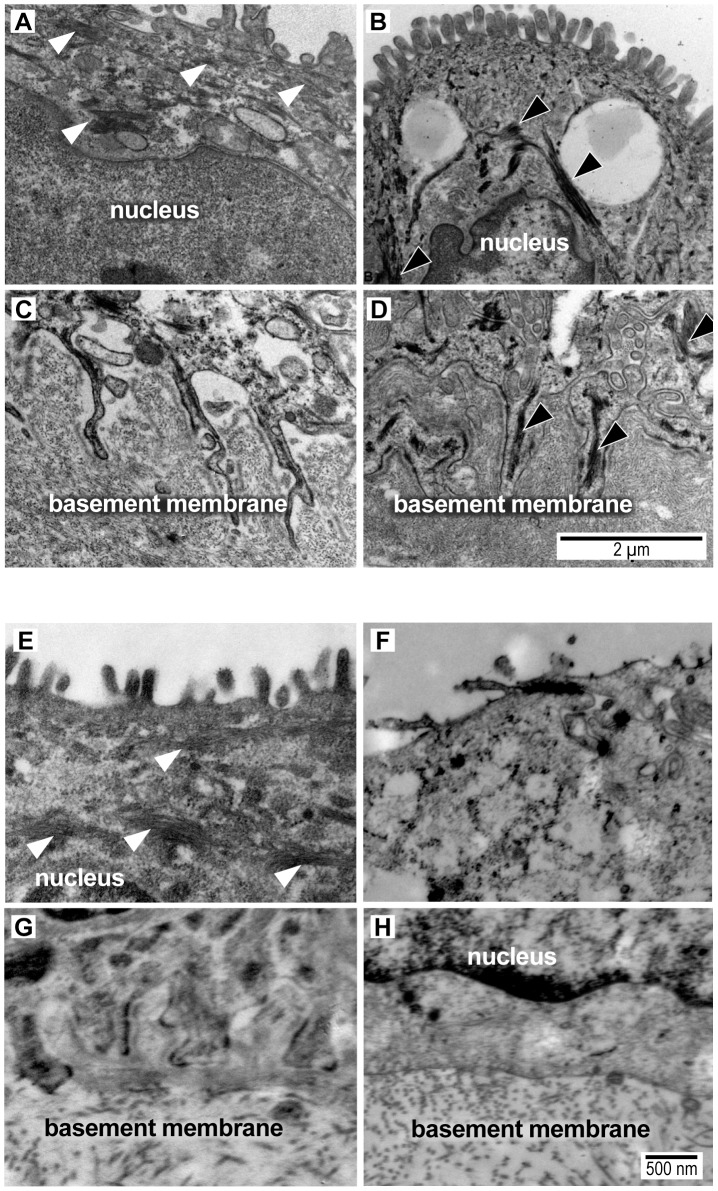
Transmission electron micrograph images of intermediate filaments (white arrowheads) in the cytoplasm and amnion foot processes from a macaque saline control (A,C) and macaque GBS sample (B,D) and from a woman at term undergoing cesarean section prior to labor (E,G) and another woman after PPROM/preterm birth (F,H). Intermediate filament aggregates (black arrowheads) are evident in the macaque GBS and human PPROM case, but not the normal controls. The basal amnion processes are broader and appear shorter with prominent central IF aggregates (black arrowheads) in the GBS-infused sample (D) and after PPROM (H). In some human PPROM cases, the basal amnion processes are nearly obliterated. Images shown were obtained from macaque (GBS-1, Saline-5) and human tissues (Control-1, PPROM-7) that correlate with the data shown in Tables S1 and S5. The measurement bar in panel D may be applied to panels A, B and C. The measurement bar in panel H may be applied to panels E, F, G and H.

As CK network dysfunction is a novel finding in amniotic epithelium after choriodecidual infection, we further investigated the amniotic epithelium ultrastructure with transmission electron microscopy in both macaque (GBS and saline; N = 2 each) and human chorioamnion (normal term, N = 6; PPROM, N = 11). Clinical information from the human pregnancies is listed in Table S5 and electron microscopy images shown in Figure 4. In some cases, GBS infection or human PPROM was associated with destruction of the amniotic epithelium or such poor histology that we could not perform electron microscopy (N = 5 PPROM cases excluded, leaving N = 6 for analysis; Table S5); therefore, statistical analysis focused on the well-preserved human samples.

We hypothesized that intermediate filaments would be retracted from the periphery leading to shortening of the amnion foot processes along the basement membrane as dysfunctional intermediate filaments retract from peripheral organizing centers. At the basal surface of the amnion, foot processes in the macaque control were thin and elongated with relatively dispersed cytoplasmic intermediate filament networks. In the GBS cases, amniocyte foot processes were much broader with prominent central intermediate filament aggregates. In human amnion obtained from normal pregnancies at term, the amniocyte foot processes were somewhat broader than in the macaque, but also demonstrated a highly folded undulating profile along the basement membrane. Human amniocyte foot processes were on average 594 nm shorter in the PPROM cases than controls, which was highly significant after controlling for processing site (p = 0.002, Table 5, Figure S4). Collectively, these studies indicate that an early choriodecidual GBS infection leads to deformation of the amniotic epithelial cell ultrastructure and CK network critical for maintaining cell structure and resisting shear stress.

**Table 5:** Amniocyte foot process length.

Measure	PPROM		Term Births	
	Mean	SE	Mean	SE
Foot Process Length at both sites combined (nm) (N_PPROM_ = 6, N_Control_ = 6)	558.9	112.6	1152.4	244.4
Foot Process Length from “Site 1” (nm) (N_PPROM_ = 1, N_Control_ = 3)	435.4	NA	621.2	105.8
Foot Process Length from “Site 2” (nm) (N_PPROM_ = 5, N_Control_ = 3)	583.7	134.6	1683.6	73.1

Human amniocyte foot processes were on average 594 nm shorter in the PPROM cases than controls, which was highly significant after controlling for processing site (p = 0.002). A histogram depicting the distribution of amniocyte foot process lengths at both sites is shown in Figure S4.

## Discussion

**Figure 5:**
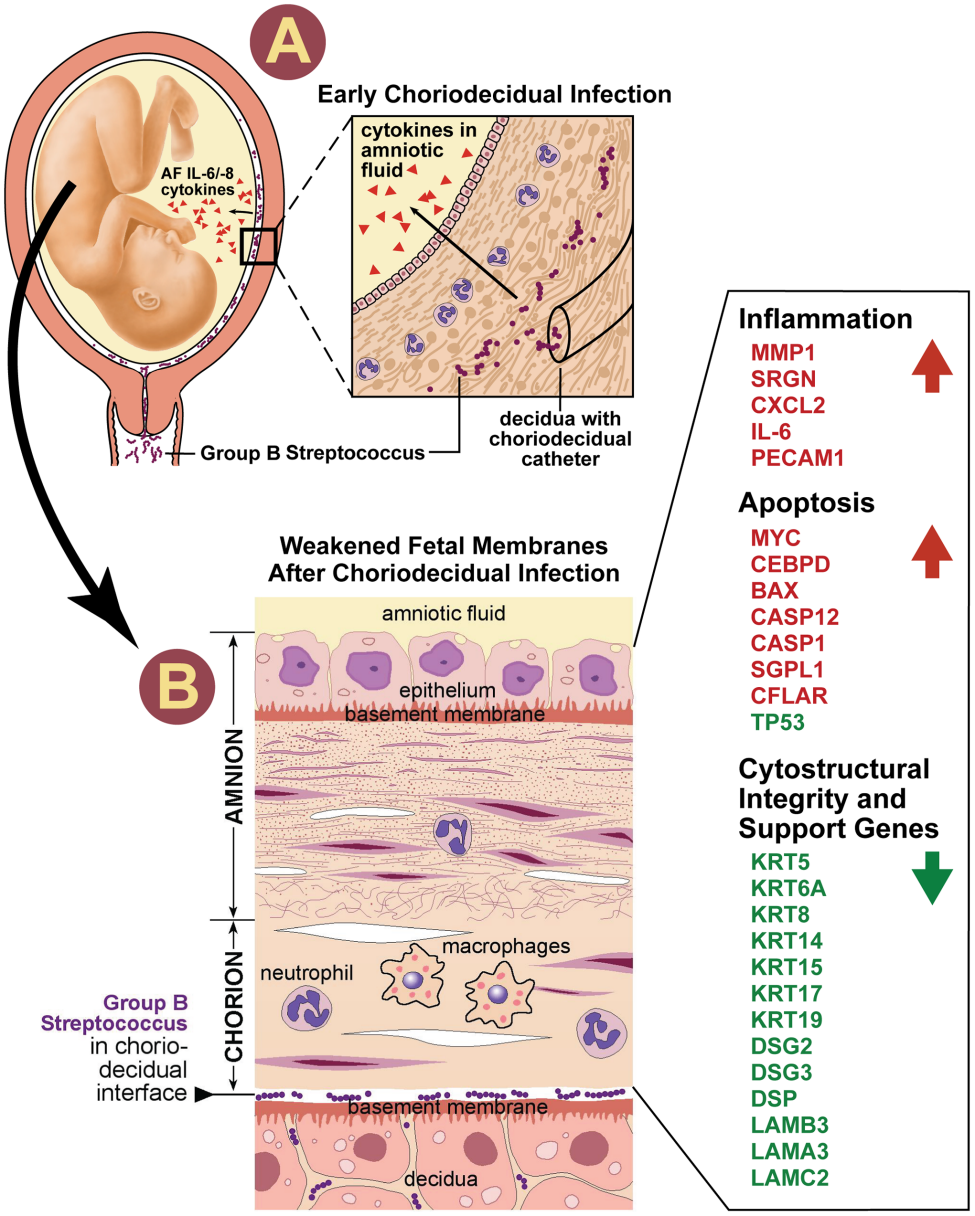
Our conceptual model of the events is shown to connect choriodecidual infection with weakening of the chorioamnion intracellular matrix. First, bacteria from the vagina traffics into the choriodecidual space (panel A). Inflammatory mediators (e.g. IL-8, IL-6, TNF-α) are produced by the decidua and/or membranes, which diffuses through the chorioamnion and into the amniotic fluid. CK synthesis becomes downregulated and intracellular pools of cytokeratins gradually become depleted. When the reserves of cytokeratins are depleted, peripheral retraction of the cytokeratin network occurs along with formation of aggresomes. Perturbations in amnion CK networks may act synergistically to increase vulnerability to membrane weakening including other pathways mediated by the pathogen, MMP, or oxidative stress. Panel B illustrates the histologic changes that begin to occur in the chorioamnion. Neutrophils infiltrate the decidua and chorion. The chorion thins and nuclei in the amniotic epithelium begin to undero apoptosis appearing pyknotic in our illustration. The side-bar lists selected differentially expressed genes identified by IPA analysis IL, Interleukin; CK, cytokeratin, MMP matrix metalloprotease. Gene symbol color indicates significantly differentially expressed up- (red) or down- (green) regulation. Note: neutrophils and macrophages are included in this conceptual model as these cell types contribute to chorioamnionitis histopathology and were observed in our results. We do not mean to infer that these are the responsible cell types for the changes observed.

Our results demonstrated a large-scale downregulation of amniocyte CK synthesis, retraction of the intermediate filament network from the basement membrane and formation of dense intermediate filament aggregates after GBS exposure or PPROM. CK are components of intermediate filaments vital for maintaining cellular structure, elasticity, and resisting shear stress. Weakening of the intermediate filament network, which occurs in several human disorders with mutated CK genes (e.g. epidermolysis bullosa simplex), increases the risk of membrane dysfunction [31]. Loss of CK also makes cells vulnerable to apoptosis, which is a typical finding in placentas following PPROM or after exposure to infection [13], [32], [33]. Prior studies in women with PPROM have focused on the contribution of matrix metalloproteinases to weakening of collagen in the extracellular matrix [12]. Our study suggests that chorioamnion weakening also occurs through a novel mechanism of downregulation of CK gene expression within the amniotic epithelium leaving the amnion vulnerable to apoptosis, shear stress, and rupture (Fig. 5). The observation that the amniotic epithelium provides the greatest tensile strength to the chorioamnion imparts even greater significance to our study [17].

Whether cytokeratin downregulation is a direct cytotoxic effect of GBS in the chorioamnion or an indirect effect of cytokine production by cells in the placenta is unknown. GBS and other gram-positive cocci are known to bind cytokeratin 8 [34]. GBS binding of cytokeratin and other extra- and intracellular matrix components (e.g. laminins, fibronectin) has been hypothesized to facilitate colonization [35] or even transmigration across epithelia, where GBS co-localized with CK expression [36]. Cytokeratin downregulation may be an indirect effect of cytokine stimulation, particularly IL-6, which has been associated with perturbations in CK synthesis and morphology *in vitro* [37]. Decreased *TP53* expression may also be an important factor in CK downregulation for at least *CK8*; transfection of lung cancer cell lines with antisense p53 cDNA results in low *CK8* expression and collapse of CK networks [26]. The remarkable breadth of CK downregulation observed following GBS exposure suggests that downregulation may be a protective host mechanism to prevent bacterial invasion of the amniotic fluid or placental colonization in the short-term.

Any benefit of CK downregulation in preventing bacterial invasion of the amniotic fluid may be short-lived and at the expense of the cytoskeletal support of the cell within several days of the initial insult [38]. Generally, *de novo* synthesis of intermediate filaments is continuous in peripheral intermediate filament organizing centers, but if CK gene transcription synthesis stops, formation of the filaments persists for some time using intracellular reserves of non-filamentous keratin precursors [30], [39]. Eventually, intracellular pools of CK become depleted and retraction of the intermediate filament network begins in the periphery. Degradation of the abnormal filaments overwhelms the ubiquitination-proteosomal system, which leads to formation of aggresomes (abnormal dense intermediate filaments) [29], [40]. We hypothesize that CK loss is a critical factor in increasing amniotic epithelial vulnerability to apoptosis, focal membrane weakening, and PPROM.

Multiple genes and pathways associated with apoptosis were upregulated in our model (e.g. *CFLAR*, *BAX*, *CASP1*, *CASP12*, and apoptotic DNA fragmentation gene set). Apoptosis is well known to occur in the chorioamnion of women with PPROM associated with infection and in the context of oxidative stress from smoking or abusing cocaine [41], [42], [43], [44], [45], [46]. GBS may directly induce apoptosis through a variety of mechanisms including cysteine proteases (calpains; GBS β-hemolysin dependent) [47], nitric oxide (oxidative stress) [48], release of cytochrome c and caspases 3 and 9 [49], and release of prokaryotic GAPDH [50]. We found upregulations of the calpain-6-like gene (*CAPN6*; log_2_ fold change 0.7), nitric oxide mediated signal transduction gene set (log_2_ fold change 0.85; p = 0.015) and smaller changes in caspase 3 and 9 expression. Caspase-3, -6, and -7 have been associated with increased type 1 CK cleavage in the setting of apoptosis [51]. As CKs are both cytoskeletal structures but also potential chemokines/matrikines, it has been proposed that programmed disposal of these insoluble proteins results in hyperphosphorlyation and collapse into “punctate inclusions of variable sizes”. We speculate the CK retractions from the periphery seen in Figure 5 represent these intermediate apoptotic stages.

Our Ingenuity Pathway Analysis also suggested a modest increase in *BAX* (Bcl-2–associated X protein) and decreased *TP53* (p53) expression, which mediate different pathways of apoptosis [51]. This contrasts with a prior PPROM study in humans, which found elevations of both p53 and BAX in the membranes [13]. This difference in activation of apoptotic pathways may reflect our use of a single pathogen (GBS) versus the probable polymicrobial colonization of the human placentas. Increased *BAX* expression and unchanged *BCL2* expression favors apoptosis due to the increased Bax∶Bcl-2 ratio; Bax-driven apoptosis can occur independently of p53 when Bax-Bax homodimers outstrip the supply of anti-apoptotic Bcl-2 [52]. Further, IPA analysis identified both TP53 and TGFB1 among top transcription factors. TGFB1 is strongly involved in regulatory processes by negatively regulating cell differentiation, proliferation, and apoptosis with effects dependent on tissue type [53], [54]. Increased TGFB1 expression has also been shown to induce apoptosis in a wide variety of tissues including gut epithelium and hepatic adipocytes [55], [56]. Data suggest the action of TGFB1 may be dependent on TP53 expression in some tissues as well [53]. Further, decreased expression of TP53 in uterine tissues is associated with preterm birth in a mouse model via activation of the COX2/PGF Synthase/PGF_2α_ pathway [57]. Our data suggest that GBS infection of the choriodecidual either directly or indirectly (via cytokines) leads to induction of apoptotic pathways through Bax and not p53 in the chorioamnion, which may then further increase CK cleavage [13], [58]. Taken together, the observed profile of TP53 expression, BAX/BCL2 ratio, and TGFB1 suggests that GBS infection of the choriodecidua leads to apoptosis and may independently increase the risk of preterm birth.

Mechanisms triggering PPROM *in utero* have been extremely difficult to study in humans because of confounding clinical variables and heterogeneous phenotypes of preterm birth. A major strength of our model is that our studies were performed *in vivo* in a nonhuman primate model that shares many key features with human pregnancy, which differ with other animal models of preterm birth (e.g. murine, sheep) [18]; findings of a perturbed CK network were also confirmed in human samples. Overall, our nonhuman primate model of cytokine elevations in the amniotic fluid without bacterial invasion reflects the clinical scenario in up to 25% of cases of human preterm labor, which makes this model highly relevant to the human condition [59], [60], [61].

Study limitations include a modest sample size, which is a necessary constraint of nonhuman primate research due to the costly nature of the experiments and for issues of conservation. These experiments are extremely expensive, which makes a study design involving prospective sampling of the chorioamnion in multiple animal groups with adequate power not possible even in the context of a large federally funded scientific grant. Two additional limitations biased our electron microscopy findings towards the null hypothesis, which makes our highly significant findings more remarkable. First, we excluded the most severe cases of PPROM due to poor histology. Secondly, as the basement membrane became flatter in our PPROM cases (with loss of CK and amniocyte foot processes), we tended to measure foot process length in areas that still had some folding of the membrane creating selection bias towards areas that appeared more normal. Overall, our findings suggest that even in the absence of AF invasion, bacteria in the choriodecidua induce an inflammatory response that is associated with downregulated expression of CK and many cytostructural elements important for chorioamnion integrity and support, which correlates with evidence of CK network dysfunction.

Changing our study design to inoculate a different organism with greater pathogenicity may have resulted in bacterial trafficking through the chorioamnion into the amniotic fluid and rapid preterm labor [62]. The GBS strain (COH1, [63]) used in our experiments belongs to the highly virulent MLST17 clone of serotype III, which is commonly associated with late-onset disease and GBS meningitis [64], [65]. In recent experiments, we have discovered that COH1 is less invasive into the chorioamnion than other GBS strains [66]. COH1 is less hemolytic than other GBS strains [e.g. A909 (Serotype Ia), NCTC10/84 (Serotype V)] and whether hemolysin expression is a major driver of GBS chorioamnion invasion is under investigation [67]. Interestingly, if bacterial trafficking through the chorioamnion had occurred, tissue damage may have been so extensive that we would have lacked the ability to discriminate subtle changes in ultrastructure associated with CK perturbations. Our model of a transient GBS infection of the choriodecidua followed by cytoskeletal weakening of the amniotic epithelium is clinically a very plausible mechanism of PPROM, which often occurs in the absence of detection of bacteria within the amniotic fluid.

Our study suggests that the loss of intermediate filament cytostructural support within the amniotic epithelium is another major contributor to chorioamnion weakening that should be considered alongside other mechanisms such as apoptosis, MMP activation, and oxidative stress. The length of time between initial infection and final depletion of the cytokeratin reserves of intermediate filaments may represent a “PPROM clock” that corresponds with the gestational length from the choriodecidual infection until membrane rupture. Future directions in understanding PPROM should focus on the relevance of CK networks to the integrity of amniotic epithelium over gestation and in the setting of infection. A better understanding of the time course of keratin assembly, disassembly and perturbations in CK networks within the amniotic epithelium may provide insight into how long choriodecidual infections are generally present prior to membrane rupture. More work is also needed to understand the regulation of cytostructural mRNA within the chorioamnion and the contribution of changes in miRNA. Therapies that stabilize CK networks and promote keratin assembly may lead to new therapeutic targets for the prevention of PPROM and preterm birth.

## Materials and Methods

Many of the methods related to our animals and study groups, uterine activity, GBS and bacterial cultures, quantitation of inflammatory mediators (cytokines, prostaglandins, matrix metalloproteinases), and performance of the microarray, qRT-PCR, microarray and IPA analysis have been previously published [20], [68].

### Ethics Statement

This study was carried out in strict accordance with the recommendations in the Guide for the Care and Use of Laboratory Animals of the National Research Council and the Weatherall report, “The use of non-human primates in research”. The protocol was approved by the Institutional Animal Care Use Committee of the University of Washington (Permit Number: 4165-01). All surgery was performed under general anesthesia and all efforts were made to minimize suffering.

Human placental samples were obtained specifically for this study under the approval of the University of Washington IRB 34004 and through the Global Alliance for the Prevention of Prematurity and Stillbirth (GAPPS, Seattle, WA, USA) Tissue Repository under the approval of Seattle Children's Hospital IRB 12879 and 13975. For both the University of Washington and GAPPS samples, subjects provided written informed consent.

### Animals and Study Groups

Ten chronically catheterized pregnant monkeys (*Macaca nemestrina*) at 118–125 days gestation (term = 172 days) received one of two experimental treatments: 1) choriodecidual and intra-amniotic saline infusions (n = 5), or 2) GBS choriodecidual inoculation (n = 5). In two saline controls, fetal samples were not collected due to either an inability to place the fetal catheter during initial surgery or clotting of the fetal catheter. This resulted in three fetal cytokine analyses in the saline group. In one GBS case, technical problems led to only intermittent data collection and so the remaining uterine activity data was excluded for this animal. The cytokine analyses were previously published and are presented to give context to the genomics analyses [20].

In our model, pregnant pigtail macaques were time-mated and fetal age determined using early ultrasound. Temperature in the animal quarters was maintained at 72–82°F. Animals were fed a commercial monkey chow, supplemented daily with fruits and vegetables and drinking water was available at all times. The tethered chronic catheter preparation was used for all *in vivo* experiments and is a major breakthrough in studying maternal-fetal immunologic responses [5], [69]. The animal was first conditioned to a nylon jacket/tether system for several weeks before surgery, which allowed free movement within the cage, but protected the catheters. On day 118–125 of pregnancy (term = 172 days) catheters were surgically implanted via laparotomy into the maternal femoral artery and vein, fetal internal jugular vein, amniotic cavity, and choriodecidual interface in the lower uterine segment (between uterine muscle and fetal membranes, external to amniotic fluid). Fetal ECG electrodes and a maternal temperature probe were also implanted. Post-operative analgesia was provided by a 25 microgram fentanyl patch applied the day prior to surgery, in addition to postoperative indomethacin. After 48 hours, the animals appeared to have recovered from surgery based on a return to baseline for activity, appetite, and bowel function.

After surgery, the animal was placed in the jacket and tether with the catheters/electrodes tracked through the tether system. Cefazolin and terbutaline sulfate were administered to reduce postoperative infection risk and uterine activity. Both cefazolin and terbutaline were stopped at least 72 hours before experimental start (∼13 half-lives for terbutaline, 40 half-lives for cefazolin, >97% of both drugs eliminated), which represented approximately a 7–10 day period of postoperative terbutaline administration. Cefazolin 1 gram was administered intravenously each day in saline controls to minimize chances of a catheter-related infection. Experiments began approximately two weeks after catheterization surgery to allow recovery (∼30–31 weeks human gestation). At our center, term gestation in the non-instrumented pigtail macaque population averages 172 days.

Intraamniotic pressure was continuously recorded, digitized, and analyzed by previously described methods [20]. The integrated area under the intrauterine pressure curve was used as the measure of uterine activity and reported as the hourly contraction area (HCA; mmHg•sec/hr) over 24 hours. Preterm labor was defined as >10,000 mmHg•sec/hr associated with a change in cervical effacement or dilation.

### Histology

After cesarean section, placenta samples underwent tissue fixation in 10% neutral buffered formalin. Complete gross and histopathologic examination was performed on placentas. H&E-stained, full-thickness paraffin sections (placental disc, umbilical cord, fetal membrane roll) were examined from each case to exclude inflammation, necrosis, fetal vascular thrombosis, or other histopathological findings. Chorioamnionitis was diagnosed by the presence of a neutrophilic infiltrate at the chorion-decidua junction (mild) or amniochorion junction (moderate or severe). Funisitis denoted neutrophils in the umbilical vessels and/or surrounding connective tissue.

### RNA Extraction and Microarray Processing

To study genetic pathways in *M. nemestrina*, we used the Affymetrix Rhesus Macaque Array (GeneChip® Rhesus Macaque Genome Array, Affymetrix, Santa Clara, CA), which allows interrogation of 47,000 *M. mulatta* transcripts and provides comprehensive transcriptome coverage. Genetic differences between *M. mulatta* and *M. nemestrina* are predicted to be <1%, which is consistent with our published data [70]. RNA extraction was performed by the CHDD Genomics Core Laboratory followed by the manufacturer's protocols using the GeneChip platform by Affymetrix. Briefly, these methods include the synthesis of first- and second-strand cDNAs, the purification of double-stranded cDNA, the synthesis of cRNA by in vitro transcription (IVT), the recovery and quantitation of biotin-labeled cRNA, the fragmentation of this cRNA and subsequent hybridization to the microarray slide, the post-hybridization washings, and the detection of the hybridized cRNAs using a streptavidin-coupled fluorescent dye. Hybridized Affymetrix arrays were scanned with an Affymetrix GeneChip 3000 fluorescent scanner. Image generation and feature extraction was performed using Affymetrix GeneChip Command Console Software.

### Single Gene Analysis

The data discussed in this publication have been deposited in NCBI's Gene Expression Omnibus (Edgar *et al.*, 2002) and are accessible through GEO Series accession numberGSE46290. Analysis of the microarray data focused first on differential expression of single genes. Raw microarray data was pre-processed and analyzed with Bioconductor (http://www.bioconductor.org/) [71]. Several quality control steps were carried out to insure that the data was of high quality: 1) visual inspection of the GCOS DAT chip images, 2) visual inspection of the chip pseudo-images generated by the Bioconductor affyPLM package, 3) generation of percent present calls and average background signals with the Bioconductor simpleaffy package, 4) generation and inspection histograms of raw signal intensities, and 5) generation and comparison of the Relative Log Expression and Normalized Unscaled Standard Errors using the Bioconductor affyPLM package. The data was normalized with the Bioconductor affy package using Robust Multiarray Averaging (RMA) [72]. From the normalized data, genes with significant evidence for differential expression were identified using the limma package in Bioconductor [73]. P-values were calculated with a modified t-test in conjunction with an empirical Bayes method to moderate the standard errors of the estimated log-fold changes. P-values were adjusted for multiplicity with the Bioconductor package qvalue, which allows for selecting statistically significant genes while controlling the estimated false discovery rate.

### Gene Set Analysis (GSA)

Next, the data was analyzed using GSA in order to investigate categories of genes [21], [74]. GSA assesses the statistical significance of pre-defined gene sets/pathways as a whole rather than of single genes, which allows for the identification of modest but concordant changes in expression of individual genes that may be missed by single gene analysis. GSA software is available as R code (http://www-stat.stanford.edu/~tibs/GSA/) [21], [75]. GSA considers all the genes in the experiment and allows for the identification of gene sets with strong cross-correlation by boosting the signal-to-noise ratio, which makes it possible to detect modest changes in gene expression. In GSA, the p-values that are calculated to test the null hypothesis are based on permutations of the sample labels. We used gene set databases for the GSA from Gene Ontology [76] (Biological Process, Molecular Function, and Cellular Component), and the functional C4 gene set from the Molecular Signature Database [75] ([75](http://www.broadinstitute.org/gsea/msigdb/index.jsp).

### IPA Analysis

We used the Ingenuity Pathway Analysis (IPA) software (Ingenuity® Systems, www.ingenuity.com) to discover pathways and transcriptional networks in the gene expression microarray data. Our data set containing gene identifiers and corresponding expression changes between the experimental groups and p-values was uploaded into the IPA application. Each identifier was mapped to its corresponding object in the Ingenuity Knowledge Base. The Functional Analysis identified the biological functions and/or diseases that were most significant to the data set. Genes from the data set with more than 1.5-fold differential expression (up/down regulation) and p<0.05 that were associated with biological functions and/or diseases in the Ingenuity Knowledge Base were considered for the analysis. The categories “Top Molecular and Cellular Functions” and “Top Upstream Regulators” were primarily used in this analysis. Right-tailed Fisher's exact test was used to calculate a p-value determining the probability that each biological function and/or disease assigned to that data set is due to chance alone. The IPA Path Designer Graphical Representation was used to generate figures based on the July 2012 IPA mapping file. Molecules are represented as nodes, and the biological relationship between two nodes is represented as an edge (line). All edges are supported by at least one reference from the literature, from a textbook, or from canonical information stored in the Ingenuity Knowledge Base. Human, mouse, and rat orthologs of a gene are stored as separate objects in the Ingenuity Knowledge Base, but are represented as a single node in the network. The intensity of the node color indicates the degree of up- (red) or down- (green) regulation. Nodes are displayed using various shapes that represent the functional class of the gene product. Edges are displayed with various labels that describe the nature of the relationship between the nodes (see figure legends for details). IPA also allows prediction of the activation or inhibition of transcription factors involved in the gene expression patterns seen in our study.

### Validation of cDNA Microarray by Quantitative RT-PCR

Quantitation of mRNA levels was performed by the CHDD Genomics Core Laboratory using fluorogenic 5′ nuclease-based assays and has been previously described [77], [78], [79].

### Immunofluorescence of Chorioamnion and Confocal Microscopy

After cesarean section, fetal membranes from placental samples for immunohistochemistry underwent freezing in OCT embedding compound, fresh-frozen and stored at −70°C. Fresh-frozen sections were serially cut with microtome to 5 micron standardized width and mounted on slides. After a 15-minute acetone fixation, slides were air dried and then permeabilized (1% Triton X-100) for 30 minutes prior to blocking (5% donkey serum) for 1 hour. Slides were then immunostained with primary rabbit anti-CK6 antiserum (1∶750, ab24646, Abcam, Cambridge MA), in an overnight incubation, and subsequently with a secondary donkey anti-rabbit 488 antibody (1∶400). Counterstaining with DAPI was performed prior to coverslip application.

Images were acquired with a FV-1000 laser scanning confocal microscope coupled to an IX-81 inverted microscope (Olympus, Center Valley, PA) and controlled by Fluoview software, version 3.1. DAPI labeling was imaged using a 405 nm laser line and 425 to 475 nm bandpass diffraction filter. Alexa488 was detected with the 488 nm line using a diffraction 500 to 550 nm filter. Images for quantitation of intensities and area were obtained with a 20×/NA0.75 UPLAPO dry objective lens, zoom of 2 and a 512×512 box size for a final pixel size of 0.621 µm. High-resolution images for qualitative analysis were collected with a 60×/NA1.35 UPLSAPO oil immersion objective, zoom of 3 and a 1024×1024 box size for a final pixel size of 0.069 µm/pixel. Images at each magnification were collected using the same laser and detector settings for all labeled specimens and negative controls.

Image analysis for CK6 content was performed with ImageJ (Rasband, W.S., ImageJ, U. S. National Institutes of Health, Bethesda, Maryland, USA, http://imagej.nih.gov/ij/, 1997–2012) running on a Mac Pro, OS10.6.8. Analysis of moderate resolution images began with background subtraction based upon the mean intensity of a selected acellular region in a representative image. This value was subtracted from all images prior to measurement. The mean intensity and standard deviation of the mean was measured in each image following background subtraction and the 2 values were summed to establish a threshold. The chorion and epithelium were manually outlined in each image. The area of regions above threshold and their summed intensities (“raw integrated density) were recorded. These operations were combined in a macro that automated background subtraction and measurements.

The high-resolution images were coded by random number generator and scored by a two blinded reviewers (KAW, RMM) for qualitative assessment of CK retraction and speckling (yes/no). CK retraction was defined as a “blunted or faded” CK staining pattern along the highly folded basement membrane of most cells. Speckling was defined as “bright spots or areas of immunofluorescence” associated with CK aggregation in some or all cells. Five images per slide were taken from random areas of the amniotic epithelium. Each image was scored with one point for retraction and one point for speckling from each reviewer. We also created a binary predictor test variable for retraction and speckling combined so that cases with both retraction and speckling were coded as 0 and other combinations coded as a 1. Results were analyzed with a logistic regression model clustered by case. One GBS case was excluded, because the chorioamnion tissues lacked amniotic epithelium; one saline case was excluded because the frozen chorioamnion could not be located. The principal component analysis and cytokine data suggested that a single GBS case appeared very similar to the saline cases and therefore we grouped a single GBS case with the saline cases for this analysis (GBS, N = 3; Saline, N = 5).

### Transmission Electron Microscopy

Formalin-fixed and paraffin-embedded tissues from macaque (GBS-1 and -2, Saline-3 and -5, Table S1) and human chorioamnion (Table S5) were used for these experiments. First, the tissues were deparaffinized and fixed in 4% paraformaldehyde and 2% glutaraldehyde in 0.1M sodium cacodylate buffer (pH 7.4) for at least 2 hours to overnight. Tissues were cut (1×4 mm^2^) and immersed in 0.1M sodium cacodylate buffer (3 changes, 10 min each or overnight). After fixation, tissues were then immersed in 1% osmium tetroxide in buffered saline. Tissues were then dehydrated with 50% ethanol (10 minutes) followed by en bloc staining with 2% uranyl acetate in70% ethanol (2 hours). Further dehydration was done in a series of alcohol dehydration steps (95% for 10 minutes, 100% for 10 minutes ×2) and 100% propylene oxide (10 minutes ×2). Embedding was performed by a 1∶1 mixture of EMBed 812 and Propylene Oxide for 1–2 hr, then 100% EMBed 812 for 2–4 hours followed by drying in an oven at 60°C for 24 hours. Embedded samples were then cut semi-thick (Leica Reichert Ultracot S, Wein, Austria) with toluidine blue stain (1–3 min). Semi-thick sections were observed under microscope for precise location for ultrathin sections. Ultrathin sections at 70 nm were collected onto grids. Grids were stained with lead citrate. Tissue samples were processed at two different sites (University of Washington, Seattle Children's Hospital) and imaged with two different electron microscopes (JEM-1230, Jeol, Peabody, MA, USA or Zeiss EM910, Carl Zeiss AG, Oberkochen, Germany).

Quantitative analysis of TEM images was performed on the human controls and PPROM cases. Amniocyte foot processes were identified along the basement membrane folds were measured for maximum length by Olympus ITEM Soft Imaging System (Münster, Germany) by an author (RPK) blinded to case-control status (Fig. S5). Twenty to forty amniocyte foot processes were measured from each case or control using 7–10 different images. A repeated measures analysis of variance was used for to take advantage of these many measurements from each case or control. The final statistical analysis controlled for differences related to the two sites of processing and imaging (e.g. differences in chemical reagents and tissue processing times).

## Supporting Information

Figure S1 Histopathology of the fetal chorioamnion and umbilical cord are shown for a saline control (A,C) and GBS-exposed with chorioamnionitis (B,D,E). In exposed animals, neutrophilic infiltration (circles) is present in the chorioamnion (B), placental disc (D), and wall of the umbilical vein (E, inset).
(TIF)

Figure S2 Heatmap of 603 out of 52,779 differentially expressed probesets with >2.0 fold change, p<0.05 in GBS vs Saline exposed animals. When probesets were matched to genes and duplicates removed, 331 out of 19448 genes were differentially expressed (>2.0 fold change, p<0.05). The black arrows indicate the GBS cases with chorioamnionitis.
(JPG)

Figure S3 Principal component (PCA) analysis of GBS cases and saline controls. PCA is a tool in exploratory data analysis and allows interpretation of variance in the data. In this figure, the PCA analysis reveals that the saline group is separated from the 5 GBS samples, but the GBS cases without chorioamnionitis are mixed in with the GBS cases with chorioamnionitis. This would indicate that the GBS cases with chorioamnionitis are too confounded with the GBS cases without chorioamnionitis to benefit from a stratified analysis.
(TIF)

Figure S4 The histogram depicts the length of foot processes at the basal amniotic epithelial surface obtained from transmission electron microscopy images from human PPROM cases (blue, n = 7) and human controls (red, n = 6) stratified by site. Human amniocyte foot processes were on average 594 nm shorter in PPROM cases (p = 0.002 after adjustment for processing site).
(TIF)

Figure S5 An example of a measurement (white bar) of an amniocyte foot process length is shown here. The black arrow indicates CK aggregation (aggresome) indicative of CK network dysfunction.
(TIF)
