# Supplementary figures and images for: Group B Streptococcal Infection of the Choriodecidua Induces Dysfunction of the Cytokeratin Network in Amniotic Epithelium: A Pathway to Membrane Weakening
Source: PLoS Pathog. 2014 Mar 6;10(3):e1003920. doi: 10.1371/journal.ppat.1003920 (PMC3946355; doi:10.1371/journal.ppat.1003920)

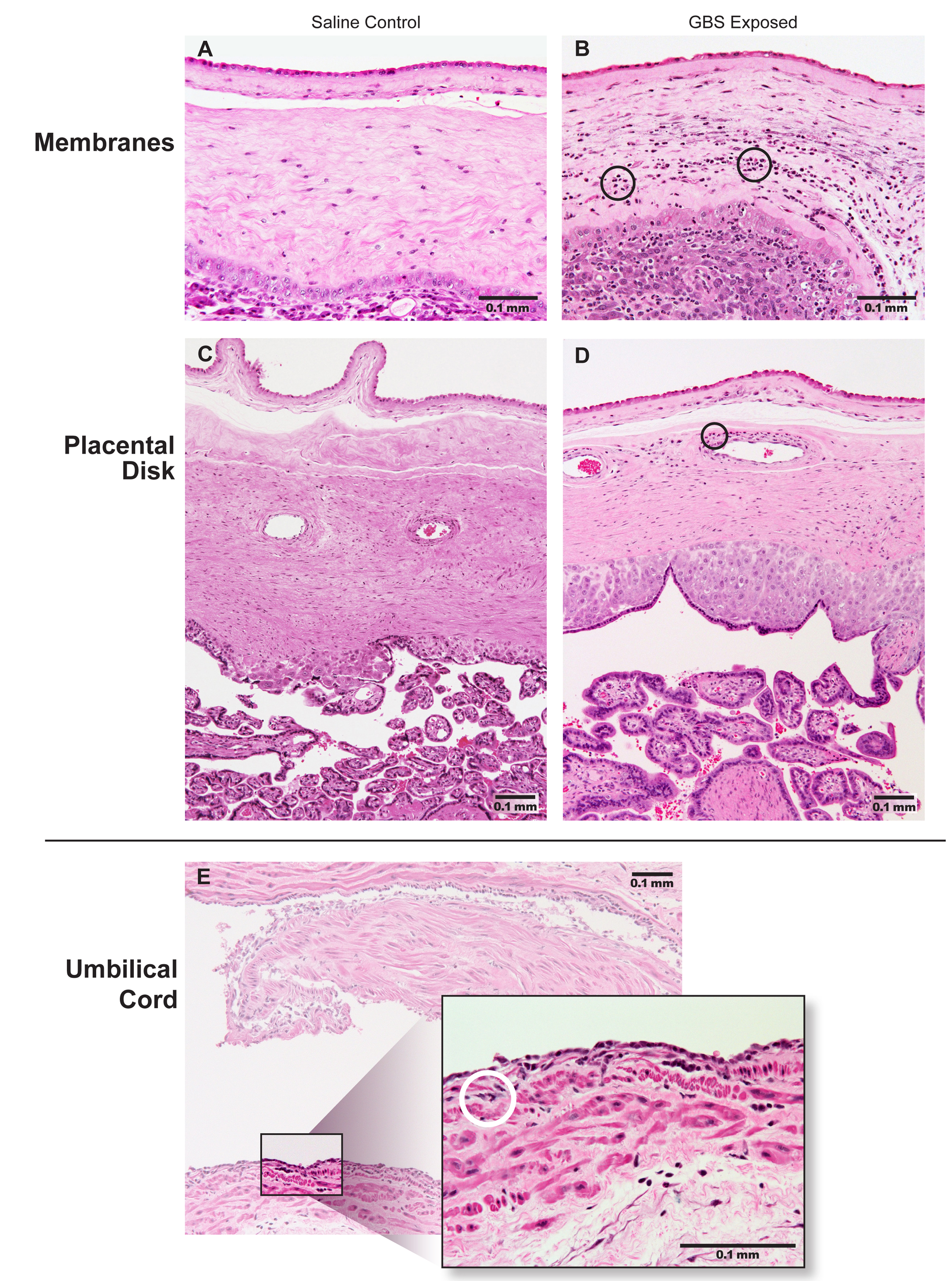

Supplement: Figure S1 — Histopathology of the fetal chorioamnion and umbilical cord are shown for a saline control (A,C) and GBS-exposed with chorioamnionitis (B,D,E). In exposed animals, neutrophilic infiltration (circles) is present in the chorioamnion (B), placental disc (D), and wall of the umbilical vein (E, inset). (TIF) [file ppat.1003920.s001.tif]

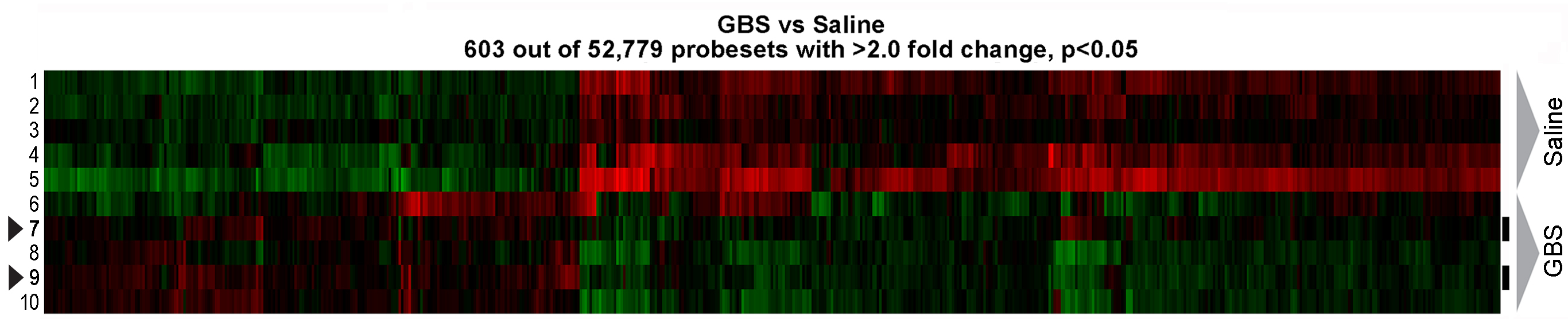

Supplement: Figure S2 — Heatmap of 603 out of 52,779 differentially expressed probesets with >2.0 fold change, p<0.05 in GBS vs Saline exposed animals. When probesets were matched to genes and duplicates removed, 331 out of 19448 genes were differentially expressed (>2.0 fold change, p<0.05). The black arrows indicate the GBS cases with chorioamnionitis. (JPG) [file ppat.1003920.s002.jpg]

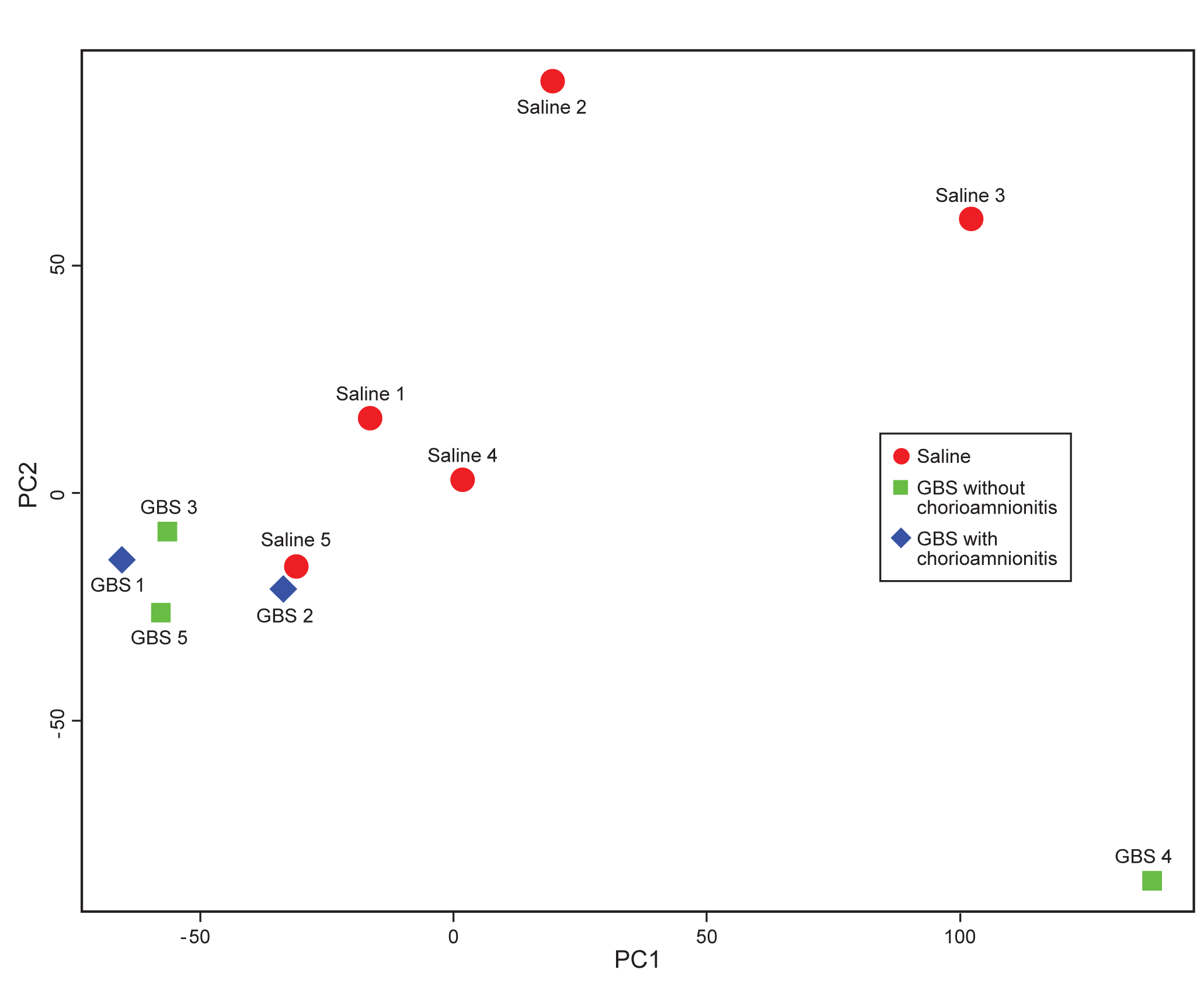

Supplement: Figure S3 — Principal component (PCA) analysis of GBS cases and saline controls. PCA is a tool in exploratory data analysis and allows interpretation of variance in the data. In this figure, the PCA analysis reveals that the saline group is separated from the 5 GBS samples, but the GBS cases without chorioamnionitis are mixed in with the GBS cases with chorioamnionitis. This would indicate that the GBS cases with chorioamnionitis are too confounded with the GBS cases without chorioamnionitis to benefit from a stratified analysis. (TIF) [file ppat.1003920.s003.tif]

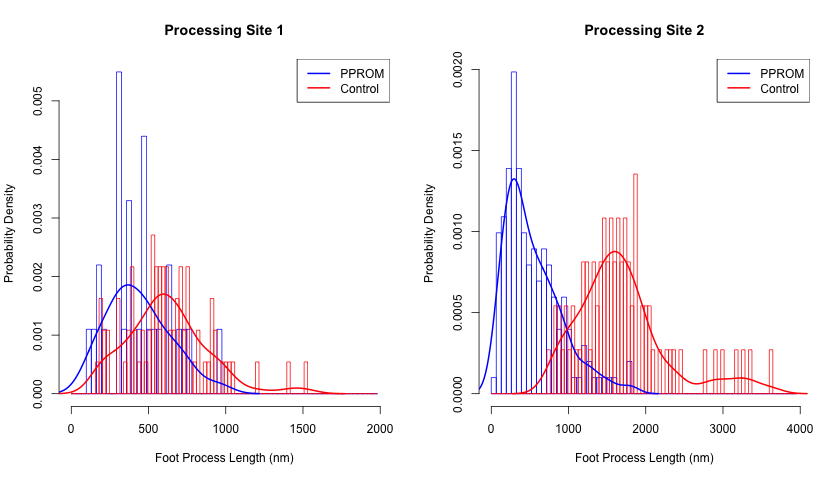

Supplement: Figure S4 — The histogram depicts the length of foot processes at the basal amniotic epithelial surface obtained from transmission electron microscopy images from human PPROM cases (blue, n = 7) and human controls (red, n = 6) stratified by site. Human amniocyte foot processes were on average 594 nm shorter in PPROM cases (p = 0.002 after adjustment for processing site). (TIF) [file ppat.1003920.s004.tif]

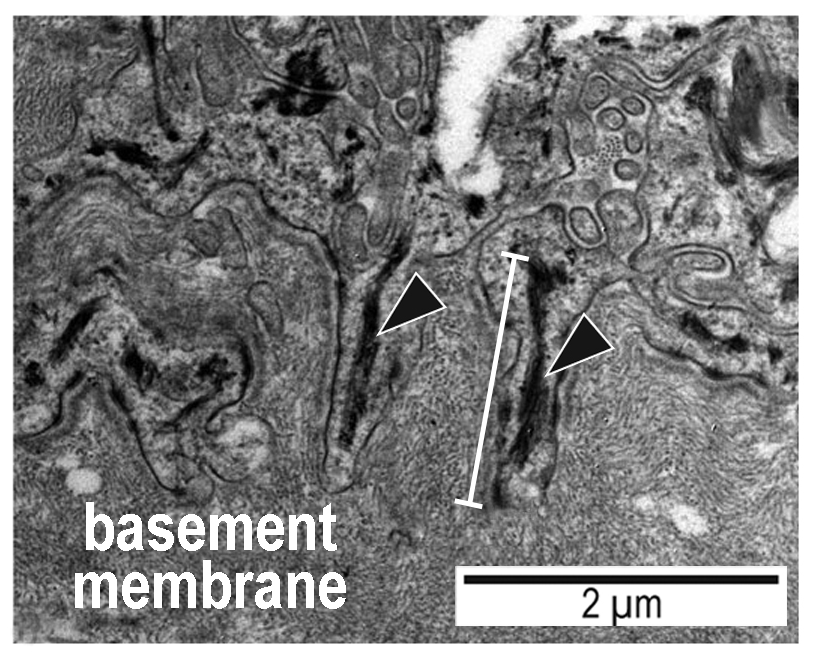

Supplement: Figure S5 — An example of a measurement (white bar) of an amniocyte foot process length is shown here. The black arrow indicates CK aggregation (aggresome) indicative of CK network dysfunction. (TIF) [file ppat.1003920.s005.tif]
